# Mechanisms and Biomarkers of Apoptosis in Liver Disease and Fibrosis

**DOI:** 10.1155/2012/648915

**Published:** 2012-04-09

**Authors:** Jayashree Bagchi Chakraborty, Fiona Oakley, Meagan J. Walsh

**Affiliations:** The Fibrosis Laboratory, Institute of Cellular Medicine, Newcastle University, Newcastle upon Tyne NE2 4HH, UK

## Abstract

Liver fibrosis and cirrhosis are a major cause of morbidity and mortality worldwide. Development of the fibrotic scar is an outcome of chronic liver diseases of varying aetiologies including alcoholic liver disease (ALD) nonalcoholic liver disease (NAFLD) including non-alcoholic steatohepatitis (NASH) viral hepatitis B and C (HBV, HCV). The critical step in the development of scar is activation of hepatic stellate cells (HSCs), which become the primary source of extracellular matrix. Aberrant apoptosis is a feature of chronic liver diseases and is associated with worsening stages of fibrosis. However, apoptosis is also the main mechanism promoting the resolution of fibrosis, and spontaneous or targeted apoptosis of HSC is associated with regression of fibrosis in animal models and patients with chronic liver disease. Given the importance of apoptosis in disease progression and resolution, there is much interest in precisely delineating the mechanisms involved and also developing biomarkers that accurately reflect the underlying pathogenesis. Here, we review the mechanisms driving apoptosis in development of liver disease and use of apoptosis -related biomarkers to aid in clinical diagnosis. Finally, we will also examine the recent literature regarding new insights into mechanisms involved in apoptosis of activated HSCs as possible method of fibrosis regression.

## 1. Introduction

Liver injury leading to fibrosis occurs in response to a variety of insults including alcohol, viral hepatitis, steatosis and insulin resistance, autoimmune disease, excessive deposition of iron or copper, and congenital abnormalities. Fibrosis is the consequence of an overactive wound healing process in response to the injury [1]. A key step in this process is activation and proliferation of HSC from periportal and perisinusoidal areas [2]. Under normal conditions, the HSC resides in the space of Disse in a quiescent phenotype storing retinoids including vitamin A [3]. Upon liver injury HSCs transform to an active phenotype, positive for alpha smooth muscle actin (*α*-SMA) and producing excessive fibrillar collagens, proinflammatory cytokines including IL-6, IL-8, MCP1, and inhibitors of matrix proteases. Initially, this process is driven by an inflammatory response and results in a controlled deposition of extracellular matrix; however, if the underlying insult persists, there is an excessive deposition of extracellular matrix including cross-linking of collagen and impairment of hepatocyte regeneration [4].

In chronic liver diseases, liver cell death is a prominent feature and correlates with worsening fibrosis [5, 6]. The cell death can occur by one of two mechanisms: necrosis or apoptosis. Apoptosis is a highly synchronised procedure requiring cellular ATP; conversely death by necrosis is ATP independent. In recent years, it has been suggested that both apoptosis and necrosis can occur in response to a single initiating factor; however, the ultimate fate of the cell is thought to depend largely on the severity of the initial damage signal. It is likely that other forms of cell death such as autophagy (self digestion) [7], paraptosis [8], necroptosis [9], and oncosis [10] also play an important role in fibrogenesis; however, in this paper, we will focus specifically on apoptosis. 

## 2. Apoptosis or Programmed Cell Death (PCD)

Apoptosis is a normal physiological process and is characterised by a well-synchronised sequence of morphological events. The dying cell undergoes nuclear and cytoplasmic condensation, blebbing of the plasma membrane, and eventually breaking apart into membrane-enclosed particles termed apoptotic bodies containing intact organelles, as well as portions of the nucleus [11, 12]. These apoptotic bodies are recognised, engulfed, and degraded by professional phagocytes, innate immune cells, and HSC [13]. Within the liver, the major cell type to eliminate apoptotic bodies is the resident liver macrophage, the Kupffer cell. In comparison, necrosis, is a pathological or accidental mode of cell death, characterised by irreversible swelling of the cytoplasm and distortion of organelles, including mitochondria [14]. Eventually there is loss of membrane integrity resulting in cell rupture and release of cellular contents. Necrosis occurs when cells are subjected to toxic stimuli such as hyperthermia, metabolic poisons, and direct cell trauma. Several important biochemical markers of apoptosis have been identified, including nuclear DNA fragmentation, activation of aspartate-specific proteases known as caspases and cell surface externalization of phosphatidylserine (PS) residues, expression of several death ligand TNFs, FasL, or overexpression of death receptors including TRADD, Fas, and DR5 [15]. Although cell death may transpire in the absence of caspases [16], the characteristic morphological features that define apoptosis are dependency of caspase activation and cleavage of specific cellular proteins or “death” substrates within the cell. Apoptosis may therefore be viewed, in biochemical terms, as a caspase-mediated form of cell death. At present, two major pathways that link apoptosis have been identified: (a) intrinsic or mitochondrial and (b) extrinsic or death receptor related.

### 2.1. Mitochondrial or Intrinsic Pathway of Apoptosis

The intrinsic pathway involves the regulation of apoptosis by mitochondria and is characterized by the release of mitochondrial intermembrane space proteins including cytochrome c, apoptosis-inducing factor (AIF), second mitochondrial activator of caspases (Smac), direct IAP binding protein with low pI (DIABLO), endonuclease G, and Omi/HtrA2 into the cytosol [17]. Cytosolic cytochrome c subsequently activates a multiprotein complex referred to as the apoptosome, which in turn leads to cleavage of procaspase-9 and downstream effector caspases (e.g., caspase-3), resulting in cell death. As such, the mitochondria have emerged as a novel target for anticancer chemotherapy. This target is based on the observation that several conventional and experimental chemotherapeutic agents promote the permeabilization of mitochondrial membranes in cancerous cells to initiate the release of apoptogenic mitochondrial proteins. This ability to engage mitochondrial-mediated apoptosis directly using chemotherapy may be responsible for overcoming aberrant apoptosis regulatory mechanisms commonly encountered in cancerous cells. Interestingly, several putative cancer chemopreventive agents also possess the ability to trigger apoptosis in transformed, premalignant, or malignant cells in vitro via mitochondrial membrane permeabilization [18]. This process may occur through the regulation of Bcl-2 family members or by the induction of the mitochondrial permeability transition. Thus, by exploiting endogenous mitochondrial-mediated apoptosis inducing mechanisms, certain chemopreventive agents may be able to block the progression of premalignant cells to malignant cells or the dissemination of malignant cells to distant organ sites as means of modulating carcinogenesis *in vivo*.

### 2.2. Death Receptor or Extrinsic Pathway of Apoptosis

The extrinsic pathway is triggered by ligation of death receptors, such as CD95 or the agonistic TRAIL receptors TRAIL-R1 and TRAIL-R2, by their cognate ligands or agonistic antibodies, which results in receptor trimerization, clustering of the receptors death domains, and recruitment of adaptor molecules (e.g., Fas-associated death domain FADD) through homophilic interaction mediated by the death domain [19]. FADD in turn recruits caspase-8 to the activated CD95 receptor to form the CD95 death-inducing signaling complex (DISC). Oligomerization of caspase-8 upon DISC formation drives its activation through self-cleavage. Caspase-8 then activates downstream effector caspases such as caspase-3 [20]. Links between the receptor and the mitochondrial pathway exist at different levels. Upon death receptor triggering, activation of caspase-8 results in cleavage of proapoptotic Bid protein, a Bcl-2 family protein with a BH3 domain only, whose truncated form is inserted into the mitochondrial outer membrane and promotes cytochrome c release and consequent activation of the apoptosome, thereby initiating a mitochondrial amplification loop [17]. In addition, cleavage of caspase-6 downstream of mitochondria can feed back to the receptor pathway by cleaving caspase-8 [21]. The idea to specifically target death receptors to trigger apoptosis in tumour cells is attractive for cancer therapy, as death receptors have a direct link to the cell death machinery. Also, apoptosis upon death receptor triggering is considered to occur independently of the p53 tumour suppressor gene, which is impaired in the majority of human tumours [22].

## 3. Apoptosis in Liver Disease

Apoptosis can occur in response to viral infection, and exposure to any kind of hepatocarcinogen, excessive alcohol consumption or due to genetic mutations. The liver resident cells express high levels of cell-death-associated receptors, for instance hepatocytes, cholangiocytes, activated stellate cells, and Kupffer cells all express Fas. High expression of the Fas receptor not only helps to maintain liver homeostasis but also helps to eliminate virally infected cells of liver by the immunocytes [23]. Fas/FasL signalling has been largely implicated in liver pathophysiology but the mitochondrial intrinsic pathways are also involved in liver homeostasis. Bid, one of the BH3 subfamily proteins, is cleaved by caspase-8, and the truncated Bid then translocates to the mitochondria where it activates the intrinsic apoptosis pathway. It has been shown that bid-deficient mice are resistant to Fas-induced hepatocellular apoptosis [24]. These studies implicate both extrinsic and intrinsic apoptotic pathways in maintaining normal liver physiology.

### 3.1. Viral Hepatitis

There are seven different types of viral hepatitis, among them hepatitis B (HBV) and hepatitis C (HCV) are the major cause of hepatic cell destruction leading to chronic hepatitis, fibrosis and increased risk of formation of hepatocellular carcinoma [25]. It is now well established that cytotoxic T cells induce apoptosis of virally infected hepatocytes via Fas/FasL and perforin-mediated pathways [26]. FasL-expressing infiltrated mononuclear cells are found in the hepatitis-C-infected patients [27]. Conversely, failure of immune cells to eliminate virally infected hepatocytes leads to viral persistence and immune surveillance causing chronic hepatitis and initiating fibrogenesis. In vivo silencing by small interfering RNA (siRNA) duplexes targeting the gene Fas protects mice from liver failure and fibrosis in two models of autoimmune hepatitis [16]. In addition to viral-induced damage, host factors such as the presence of steatosis are associated with increased hepatocyte apoptosis in patients with HCV infection. Additionally, hepatocyte apoptosis in patients with steatosis correlates with grade of fibrosis, implicating a direct link between apoptosis and fibrosis [28].

### 3.2. Alcoholic Steatohepatitis

Excessive alcohol consumption may lead to changes in fat metabolism, cause chronic inflammation, and over time promote the development of a chronic hepatitis and fibrosis called alcoholic steatohepatitis (ASH) [29]. Hepatocyte ballooning and apoptotic hepatocytes are the common features in the liver of patients with ASH [30]. Excessive hepatocyte apoptosis stimulates inflammation and results in the production of proinflammatory cytokines and reactive oxygen species by innate immune cells [31]. Additionally, oxidative stress-induced hepatocyte apoptosis is one of the consequences of acute alcohol injury [32]. Defects in endocytosis leading to accumulation of apoptotic bodies followed by inflammation may also play an important role in alcoholic liver injury. It has been found that in ethanol induced impairment receptor mediated endocytosis model defects in asialoglycoprotein receptor, which lead to defective uptake of apoptotic bodies causing severe liver injury [33]. CYP2E1 is one of the key markers upregulated in alcoholic liver disease [34]. CYP2E1 is an isoform of cytochrome p450 related to free radical generation. Increased free radical production causes DNA damage and lipid peroxidation increasing the severity of the disease by inducing oxidative stress induced hepatocytic damage [35].

### 3.3. NAFLD and NASH

Apoptotic death of hepatocytes is a common feature of nonalcoholic steatohepatitis and is associated with fibrosis [5]. Increased expression of death receptors like Fas and TNF-R has been found in most of the NASH patients. Both extrinsic and intrinsic apoptotic pathways are involved in NASH-induced hepatocyte death [36, 37]. Caspase-3 and caspase-7 are activated with disease progression and subsequently cleave a major filamentous protein known as cytokeratin-18 (CK-18) [38]. Endoplasmic reticulum stress-induced apoptosis is a feature of multiple diseases including cystic fibrosis, diabetes, and Parkinson's and prion-related diseases [39] and may be an important mechanism in NAFLD and NASH. The presence of hepatic steatosis is associated with an increase in ER stress and a subsequent increase in hepatocyte apoptosis and liver injury [40].

### 3.4. Liver Fibrosis

Liver fibrosis is the consequence of chronic liver injury. After toxic exposure hepatocytes undergo apoptosis and hepatic stellate cells migrate to the site of injury to engulf the apoptotic bodies. This engulfment promotes activation of the hepatic stellate cells to hepatic myofibroblasts, and in their activated state these cells promote deposition of extracellular matrix and scar formation in the liver. Recently it has been demonstrated that hepatocyte-specific disruption of Bcl-xL induces continuous hepatocyte apoptosis and fibrogenesis [41]. Several proinflammatory cytokines including IL-6, TNF-*α* induce unresolved hepatocytic inflammation. The profibrogenic cytokine TGF-*β* is secreted by the immune cells gathered at site of injury to phagocytose the apoptotic bodies, further fuelling the inflammatory and fibrogenic reaction [42].

### 3.5. Hepatocellular Carcinoma

Hepatocellular carcinoma (HCC) is the consequence of exposure to carcinogen or environmental pollutants, chronic viral infection, and obesity and is the 3rd major cause of cancer death worldwide. HCC is a slow progressing disease. During the initiation phase of this disease the balance between apoptosis and cell proliferation of hepatic cells is disrupted and favours proliferation, whereas hepatocytes undergo high levels of hepatocytic cell death. In response to this injury, innate immune cells migrate to the site of damage and release a plethora of proinflammatory cytokines and free radicals generating an inflammatory microenvironment, which promotes cancer progression. After chronic exposure to rounds of liver injury and inflammation hepatocytes develop mechanisms to evade apoptotic death; this results in the accumulation of damaged hepatocytes that eventually become HCC. These mechanisms include the persistent downregulation of proapoptotic molecules and upregulation of antiapoptotic proteins. Fas receptor and Fas ligand are highly expressed on hepatocytes; however, levels of these proteins are diminished during the disease progression [43]. Concurrently, decreased expression of other downstream molecules from the Fas family including FADD (Fas Associated death domain) and FLICE have been observed during HCC development [44]. Loss of other death receptors including TRAIL-R has also been linked to neoplastic growth and reduced apoptosis in HCC [45]. Antiapoptotic factor, brain and reproductive organ-expressed protein (BRE), is a death-receptor-associated protein and is upregulated in HCC. BRE binds to tumor necrosis factor receptor-1 and Fas, and in cell lines it has been shown to attenuate apoptosis by inhibiting t-Bid-induced activation of the mitochondrial pathway [46]. Normal liver homeostasis is maintained by a balance of proapoptotic and antiapoptotic genes but in the majority of cases of HCC it has been shown that there is an overexpression of antiapoptotic genes. This imbalance can be caused by different mechanisms. For example, Otsuka et al. reported that the hepatitis C virus inhibits apoptosis by overexpressing Bcl-xL [47]. One of the important antiapoptotic proteins XIAP (inhibitor of caspases) helps to bypass apoptotic pathway in HCC progression [48]. Growth arrest DNA damage-inducible gene 45*β* (GADD45beta) regulates apoptotic cell death in response to DNA damage. Downregulation of GADD45beta has been observed in HCC [49]. Another important regulator of liver cancer progression is the tumour suppressor gene p53. This gene is activated when there is DNA damage, but, in most cases of HCC, the p53 gene is mutated. Kraus and colleagues postulated that this was a result of oxidative stress and that this provided a link between chronic inflammation and genomic changes observed in precancerous cells [50].

## 4. Biomarkers of Apoptosis in Liver Disease

One of the most promising biomarkers of apoptosis is CK-18. Serum levels of CK-18 are markedly increased in patients with NASH compared with patients with steatosis or normal biopsies [51, 52]. Additionally, serum levels of uncleaved CK-18 are able to distinguish between simple steatosis and NASH. More recently, in a prospective study examining the utility of apoptosis biomarkers to predict fibrosis in patients with NASH, both full length and caspase-cleaved CK-18 were able to discriminate different stages of fibrosis with healthy controls [53]. Patients with HCV infections also have higher levels of serum CK-18 correlating with serum transferase activity; however, some patients had elevated serum CK-18 with normal transaminases, suggesting that CK-18 may be an earlier maker of liver damage. The utility of serum CK-18 levels in discriminating different stages of HCV and ALD, for example, needs to be studied further but it may provide a minimally invasive method of assessing the underlying liver injury.

## 5. Increased Apoptosis of Hepatocytes May Directly Contribute to Fibrogenesis

There is mounting evidence that suggests phagocytosis of apoptotic bodies by hepatic stellate cells may directly stimulate fibrogenesis [54]. Although liver macrophages are thought to be the main cell involved in phagocytosis, endothelial cells and fibroblasts have been demonstrated to clear apoptotic bodies [42, 54]. Both HSCs and Kupffer cells (KC) express the phosphatidyl serine receptor suggesting that both cell types are able to internalize apoptotic bodies. Engulfment of apoptotic bodies by KC is associated with a marked increase in profibrogenic factors including TRAIL, TNF-*α*, FasL, and TGF*β*1 mRNA expression 24 hours following exposure to apoptotic bodies derived from hepatocytes. Kupffer cells isolated from bile duct ligated (BDL) mice also show an increase in the expression of TRAIL-associated ligands compared with sham-operated animals. Additionally, depletion of KC from BDL mice was associated with a significant reduction in hepatocyte apoptosis and liver injury and a concurrent reduction in *α*-SMA and col1A1 mRNA expression [42]. Increase in the expression of TRAIL markers on KC may be suitable biomarkers for pathogenic hepatocyte apoptosis. Subsequent perpetuation of hepatocyte apoptosis is associated with an increase in inflammation and liver fibrosis. This suggests that engulfment of apoptotic bodies may prolong the cycle of liver injury and that targeting of KCs maybe a viable therapeutic option in cholestasis to reduce liver fibrosis.

Phagocytosis of hepatocyte-derived apoptotic bodies by stellate cells has also been implicated in fibrogenesis. Apoptotic bodies express phosphatidyl serine, which acts as an engulfment signal. Canbay and colleagues found that Lx-1 cells (human hepatic stellate cell line) express the phosphatidyl serine (PS) receptor and that these cells show a significant increase in TGF-*β*1 and Col1a1 mRNA expression after 48-hour incubation with apoptotic bodies. Blocking of engulfment of apoptotic bodies by Nocodazole abrogates the further increase in *α*-SMA and TGF-*β*1 suggesting that the increase in fibrogenic nature of the HSC was specifically as a consequence of apoptotic body engulfment [54].

## 6. Apoptosis and Reversal of Liver Fibrosis

### 6.1. Clinical Evidence for Reversal of Fibrosis Apoptosis

Until recently, transplantation was considered the only viable treatment option for cirrhosis and severe forms of liver disease [55]. However, there is a growing body of clinical evidence that suggests fibrosis is somewhat reversible. Successful treatment of underlying viral infection in patients with HCV and HBV is associated with regression of liver fibrosis and, in some instances, reversal of cirrhosis upon liver biopsy [56–58]. Similarly in cholestatic-type liver diseases, regression of liver fibrosis was reported after biliary drainage in patients with stenosis of the common bile duct. Additionally, abstinence from alcohol [59] and weight loss have also been associated with an improvement in histological analysis of fibrosis.

### 6.2. Experimental Evidence for Spontaneous Apoptosis-Mediated Regression of Apoptosis

**Figure 1:**
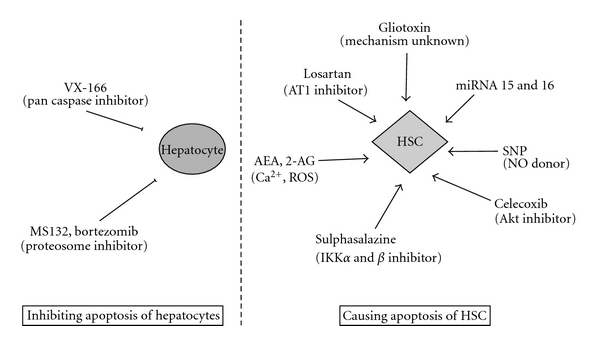
Mechanisms for targeting apoptosis to treat liver disease and cause fibrosis regression.

Summarised in Figure 1 there is a considerable experimental evidence suggesting that apoptosis of activated HSCs is the main mechanism associated with regression of fibrosis. There are two seminal papers that show spontaneous regression of fibrosis mediated by apoptosis. Firstly, Iredale and colleagues discovered that in rats treated with carbon tetrachloride to induce significant liver fibrosis, *α*-SMA-positive cells, fibrosis and hydroxy proline content returned to an almost histologically normal state 28 days following cessation of liver injury. Importantly, dual staining for TUNEL and *α*-SMA showed spontaneous resolution of fibrosis was associated with an increase in apoptosis of nonparenchymal *α*-SMA-positive cells [60]. This hypothesis has been subsequently confirmed in an additional *in vivo* model of fibrosis. Issa and colleagues ligated the bile duct of rats for 21 days to induce fibrosis, then biliodigestive anastomosis was performed and rats were allowed to recover for 45 days. There was a 5-fold decrease in *α*-SMA-positive myofibroblasts with rapid apoptosis indicated by TUNEL-positive cells two days after anastomosis [61].

This method of deleting collagen-producing HSCs has been exploited in animal models to reduce fibrosis. The potent fungal metabolic toxin, Gliotoxin, induces apoptosis of activated rat HSCs *in vitro* and *in vivo* and this is associated with a significant reduction in the number of activated HSCs and a reduction in the thickness of the bridging fibrosis and serum ALT without impairment of hepatocyte regeneration [62]. While HSCs are sensitive to apoptosis at low concentrations of Gliotoxin (1.5 uM), hepatocytes are resistant to apoptosis however they will undergo necrosis at high concentrations. Originally thought to act through an NF-kB-dependent mechanism, it is likely that Gliotoxin induces apoptosis via a rapid accumulation of glutathione, which is abrogated by pretreatment with thiol redox active agents such as PDTC [63].

There is mounting evidence suggesting that directly targeting NF-*κ*B is a viable therapeutic option in liver disease. Activated HSCs have high levels of NF-*κ*B activity and an increase in basal expression of NF-*κ*B-regulated antiapoptotic proteins including IL-6, Bcl-2 family members and GADD45*β* and A20. Blunt inhibition of NF-*κ*B-regulated antiapoptotic genes by treatment with a proteosome inhibitor prevented induction of antiapoptotic genes and caused apoptosis. Treatment of BDL mice with bortezomib was associated with a reduction in *α*-SMA-positive HSCs and fibrosis [64]. IKK2 inhibition with pharmacological agent AS602868 is also associated with attenuated fibrosis progression in mice with dietary-induced NASH and thought to work by limiting the accumulation of hepatic steatosis and hepatocyte apoptosis [65]. Sulphasalazine is a drug used commonly in humans for treatment of inflammation-type disorders including rheumatoid arthritis and inflammatory bowel disease [66]. Sulphasalazine acts to block NF-*κ*B activity by blocking the activity of inhibitor of *κ*B (IkB) kinases *α* and *β* (IKK*α* and IKK*β*) resulting in a downregulation of NF-*κ*B targets. *In vivo* administration of sulphasalazine to rats with CCl_4_-induced liver injury was associated with stimulation of HSC apoptosis (and NF-*κ*B-regulated antiapoptotic gene GADD45*β*) and a concurrent reduction in *α*-SMA-positive myofibroblasts and Col1a1 [67].

Novo and colleagues noted in cirrhotic human liver that Bcl-2 was highly expressed in *α*-SMA-positive myofibroblasts, and this staining was particularly strong in areas at the interface between fibrotic septa and regenerative nodule [68]. Upstream from Bcl-2, constitutive expression of P-Ser^536^-RelA is a key feature of activated human HSCs and is in part regulated in an autocrine fashion by angiotensin II. Inhibition of the angiotensin II receptor (AT1) and/or treatment with ACE inhibitor Captopril was associated with loss of P-Ser^536^-positive HSCs, a reduction in *α*-SMA-positive HSCs, and fibrosis regression. Importantly, in patients with hepatitis C viral infection who upon liver biopsy were found to have constitutive expression of P-Ser^536^, treatment with Losartan was associated with regression of fibrosis. Those patients who did not have P-Ser^536^ expressed on biopsy were not sensitive to Losartan therapy and did not undergo regression of fibrosis. Constitutive expression of P-Ser^536^ may be an important tissue biomarker to assess whether a patient is likely to respond to similar therapy.

Directly targeting hepatocyte apoptosis to reduce liver fibrosis may also be a viable strategy. Male db/db mice fed a methionine/choline-deficient diet (MCD) to induce NASH and liver fibrosis and subsequently treated with a pan-caspase inhibitor, VX-166, showed a reduction in *α*-SMA-positive HSCs and had reduced expression of Col1a1 mRNA and reduced fibrosis, confirmed with Sirius red staining. However, serum ALT levels were similar in mice fed MCD alone, suggesting that there was no improvement in liver injury despite some reduction in steatosis, reduction in TNF-*α* production, and formation of nitrotyrosine adducts [69]. Treatment of an injured liver (especially in the presence of ongoing injury) with an apoptosis inhibitor may be detrimental and prevent the normal removal of premalignant hepatocytes and profibrogenic myofibroblasts.

## 7. Paracrine-Mediated Apoptosis of HSC

Under normal conditions, nitric oxide exerts a paracrine effect on HSC; however, in incidences of severe liver fibrosis and cirrhosis eNOS generation is impaired. This is associated with the typical features of liver disease. *In vitro*, NO donor SNP is associated with an increase in primary rat HSC apoptosis, and overexpression of NO in Lx2 cells sensitized them to apoptosis caused by stimulation with TRAIL [70]. However, the mechanism by which apoptosis occurs is unclear. Langer and colleagues found a significant decrease in mitochondrial membrane potential (ΔΨ_*m*_) after treatment with SNP, which was not abrogated by pretreatment with pan-caspase inhibitors. It is possible that NO-mediated nitrosylation of the active site of caspase-3 impairs cleavage resulting in caspase independent apoptosis [16]. Enhancing the intracellular oxidative stress further potentiated apoptosis induced by SNP. Importantly, SNP is already approved for use in human, and we await further in vivo data to determine if HSC apoptosis results in regression of fibrogenesis.

## 8. miRNAs

miRNAs are small noncoding RNAs of 21–25 nucleotide bases thought to regulate gene transcription posttranslationally by changing the stability of mRNA through binding to the 3′ UTR [71]. (mechanism reviewed extensively in He, Nature Reviews Genetics 2004) [72]. Activation of HSCs is associated with a change in the expression profile of mRNAs including miR-16, -15b, -122, -128, -143, and -140 [73]. From this expression profiling, Guo and colleagues then identified a critical role for miR-16 and miR-15b in apoptosis. Transfection of miR-16 and miR-15b into activated HSCs concurrently reduced Bcl2 expression and increased expression of caspase-3, 8, and 9, and this was accompanied by a subsequent increase in apoptosis in activated HSCs [73]. Additionally, transfection of activated HSCs with miR-16 was associated with a reduction in cyclin D1. This was paralleled by a subsequent reduction in proliferation and increased apoptosis [73]. miR-29b is inducible in Lx2 cells by IFN-*α* in a dose-dependent fashion and was found to suppress Col1a1. This may be part of the mechanism whereby patients with HCV show a regression of fibrosis after treatment with IFN-*α*. Similarly, inhibition of 27a and 27b has been shown to aid the return of HSC to a lipid-containing phenotype by reducing the expression of retinoid X receptor *α* and was also associated with a subsequent decrease in proliferation. However, Col1a1 and *α*-SMA expression levels did not change, nor was there any significant induction of apoptosis [74]. Venugopal and colleagues found a downregulation of miR-150 and miR-194 in hepatic stellate cells isolated from the fibrotic livers from BDL rats compared with sham operated animals. *In vitro* overexpression of miR-150 and miR-194 caused decreased stellate cell activation, inhibition of cell proliferation, and reduction in *α*-SMA expression and Col1a1 levels, possibly by inhibition of c-myb (miR-150) and rac (miR-194); however, there was no significant increase in apoptosis [75]. Similarly, a decrease in miR-29 expression level is associated with Col1a1 accumulation in activated HSCs. Targeting miRNAs may provide a mechanism to cause apoptosis of activated HSCs. As yet, no serum biomarkers are available to measure miRNA activity; however, tissue biomarkers may be of use to determine if HSCs are reprogrammed to avoid apoptosis. 

## 9. TLR 9 Activation

The injured liver is exposed to high levels of danger-associated molecular patterns (DAMPs) that act as stimuli for members of the Toll-like receptor (TLR) family. Wantanabe et al. hypothesised that debris from apoptotic hepatocytes could modulate the activation of HSC via TLR9 signalling. They showed that expression of the profibrogenic genes Col1a1 and TGF*β*1 in LX2 cells and primary mouse HSCs was increased upon exposure to hepatocyte DNA or cytidine-phosphate-guanosine (CPG) oligonucleotides, the ligand for TLR9. Administration of a TLR9 antagonist prevented induction of these genes and also inhibited platelet-derived-growth-factor (PDGF) dependent HSC chemotaxis, an effect also observed in HSCs deficient for either TLR9 or its downstream adaptor molecule MyD88. The authors concluded that DNA released from damaged hepatocytes acts as a signal to “halt” HSC migration, retaining them at the site of injury and promote scar formation [76]. TLR9-mediated activation of HSCs may confer resistance to apoptosis and further promoting fibrogenesis. The role of TLR9 and DAMPS in liver disease needs further investigation.

## 10. Extracellular Matrix

The link between extracellular matrix and apoptosis of HSCs has previously been reviewed extensively; see Elsharkaway Apoptosis 2005 [77] and *Benyon Seminars *in* Liver Disease *2001 [78]. In brief, increased activity in matrix degradation enzymes is a key step in resolution of fibrosis [56]. *In vitro* treatment of HSCs with recombinant MMP9 stimulates apoptosis [79], which can be abrogated by pretreatment with an inhibitor [80]. Additionally, under conditions of recovery, interstitial MMPs have an increased collagenolytic activity, degrading the extracellular matrix and leaving the HSC more susceptible to undergo apoptosis [81]. Overexpression of MMP inhibitor TIMP-1 in mice treated with CCl_4_, was associated with an inability to undergo apoptosis of activated HSC and no concurrent resolution of fibrosis [82]. The mechanism of action by which TIMP-1 acts to inhibit apoptosis is via activating phosphatidylinositol 3-kinase and ERKs resulting in downregulation of caspases [83]. These studies suggest that in addition to secreting Col1A1, activated HSCs also secrete matrix-related enzymes that act to protect the HSC against proapoptotic signals. Serum levels of MMPs and TIMPs on their own and in combination with liver stiffness tests, enhanced liver fibrosis (ELF), and so forth are useful biomarkers to predict the existence and extent of liver disease and are a useful tool for monitoring resolution. TIMP-1 along with hyaluronic acid (HA) and the N-terminal pro-peptide of collagen type III are the key serum markers of underlying fibrosis on the ELF panel. This panel has been validated in a large cohort of patients with NAFLD and NASH [84, 85] and has been reported to have better diagnostic capabilities than other standard panel of biomarkers including MELD and the Mayo Risk (R) score in instances of primary biliary cirrhosis [86]. To date, most research has been centered around the use of biomarkers in instances of chronic liver disease; however, a recent publication by Dechene and colleagues suggests that they may have some utility in acute liver failure (ALF). Both TIMP-1 (4.2-fold) and TIMP-2 (1.6-fold) were found to be significantly increased in the sera of ALF patients compared with control individuals. Additionally, in the group of patients with ALF, MMP-1 and MMP-2 were significantly upregulated more than two fold, suggesting that these biomarkers of apoptosis and protease activity may be a useful indicator of underlying fibrogenesis. The increase in serum markers was paralleled by an increase in Col1a1 and *α*-SMA observed on liver biopsy. This research also identified a correlation between serum biomarkers of apoptosis (TIMP-1 and M65) and liver stiffness measured by FibroScan. Additionally, over a one-week observation period, there was a reduction in liver stiffness that corresponded to a reduction in serum markers of apoptosis and fibrogenesis [87]. These studies highlight the important relationship between extracellular matrix and apoptosis identifying fibrogenesis and the potential for using apoptosis biomarkers as part of a panel of markers to longitudinally monitor activity during recovery period. 

## 11. Endogenous Cannabinoid Receptors

Lipidic cannabinoid ligands and receptors CB1 and CB2 have an important role in the pathogenesis of chronic liver injury. Under normal conditions, the endocannabinoid receptors are undetectable; however, expression is slowly increased upon stellate cell activation and remains elevated in later stages of liver disease [88–90]. CB2 receptors are located in HSCs; however, CB1 receptors are also upregulated in vascular endothelium [90]. Protein expression of the CB1 receptor is increased in cirrhotic livers compared with normal human liver and appears to be expressed in nonparenchymal cells located proximal to the fibrotic septa. Immunohistochemical analysis of CB1 receptor showed co-localization with *α*-SMA-positive cells and activated cultured myofibroblasts express higher levels of the receptor compared with quiescent. In mice injured with CCl_4_ or who underwent BDL, selective inhibition of CB1 with antagonist SR141716A decreases TGF*β* and *α*-SMA expression during injury, and this was accompanied by a 37% and 41% decrease in fibrosis respectively. Additionally, in cannabinoid receptor 1 knockout mice (Cnr1^−/−^), there was a 30% and 35% reduction in fibrosis area in thioacetamide and BDL models of fibrosis. In culture, Cnr1^−/−^ HSC are more susceptible to apoptosis mediated by serum deprivation, and there was a 64% increase in apoptosis of *α*-SMA-positive cells in these animals. 

Endocannabinoids mediate apoptosis through CB1, CB2, and also transient receptor potential vanilloid 1 (TRPV1) which acts as the receptor for anandamide (AEA). The mechanism of action of AEA is well understood and reviewed more extensively by Siegmund et al., 2008. HSCs but importantly not hepatocytes are sensitive to apoptosis by both endocannabinoids AEA and 2-AG. Anandamide is the main endogenous agonist against a group of lipid mediators termed endocannabinoid and acts on the cannabinoid receptor CB1 and CB2. Anandamide (AEA) selectively kills HSC by necrosis but not hepatocytes, and this occurs independently of CB1 and CB2 and VR1 receptors. Siegmund and colleagues noted that treatment of activated HSCs with AEA induced necrosis via a Ca2+- and ROS-dependent fashion and that pretreatment with glutathione or a Ca2+ chelator (EDTA, BAPTA-tetrapotassium salt, or BAPTA-AM) significantly abrogated the effects of AEA on HSC necrosis. Additionally, preincubation with membrane cholesterol depleting agent prevented necrosis, suggesting that AEA may not be specific to CB receptors. While causing necrosis of HSC, hepatocytes appear not to be susceptible. This compound needs assessing in vivo and may prove to be a valuable therapeutic to target fibrosis [91].

## 12. Conclusions

Significant progress has been made in our understanding of the mechanisms of apoptosis and the relative contribution apoptosis plays in disease progression. Pathophysiological role of apoptosis is implicated in a number of liver diseases and contributes directly to fibrogenesis. Serum biomarkers of hepatocyte apoptosis have been well characterized in NAFLD and NASH and provide insight into disease severity. These markers, particularly cleaved CK-18, prove to be sensitive enough to distinguish between patients with simple steatosis and more advanced stages of disease. Further clinical studies are needed to determine CK-18 utility in other liver diseases. While the prospect of treating patients with liver disease with a pharmacological agent to cause the regression of fibrosis is exciting, further investigation is required into the long-term-efficacy. Blanket inhibition of hepatocyte apoptosis with pan-caspase or proteosome inhibitors reduces fibrosis; however, it may not improve liver function. Further studies are needed to determine the long-term effect of these inhibitors on liver function. Inhibition of the normal physiological process of removing damaged hepatocytes may prove to be detrimental and leave patients susceptible to developing hepatocellular carcinomas.

Ideally, next-generation antifibrotic therapies will target apoptosis-inducing mechanisms specific for activated HSC or benefit from targeted delivery systems. New insights into mechanisms of apoptosis have highlighted HSC specific involvement of NF-*κ*B signaling, cannabinoid receptor signaling, and miRNAs important in regulating apoptosis and may provide further pharmacological targets. Perhaps most importantly, HSC-specific biomarkers of apoptosis may not only provide further clinically relevant information regarding underlying disease but also predict the likelihood of a patient's response to therapy.
